# Supplementary material for: Eph-ephrin signaling couples endothelial cell sorting and arterial specification
Source: Nat Commun. 2024 Apr 3;15:2539. doi: 10.1038/s41467-024-46300-0 (PMC10991410; doi:10.1038/s41467-024-46300-0)
Supplement: Supplementary file 1 — Supplementary Information [file 41467_2024_46300_MOESM1_ESM.pdf]

# Supplementary Information

## Eph-ephrin signaling couples endothelial cell sorting and arterial specification

Jonas Stewen[1], Kai Kruse[1,2], Anca T. Godoi-Filip[1], Zenia[1], Hyun-Woo Jeong[1,3], Susanne Adams[1], Frank Berkenfeld[1], Martin Stehling[4], Kristy Red-Horse[5,6,7], Ralf H. Adams[1,#] and Mara E. Pitulescu[1,#]

1 Department of Tissue Morphogenesis, Max Planck Institute for Molecular Biomedicine, D-48149, Münster, Germany
2 Bioinformatics Service Unit, Max Planck Institute for Molecular Biomedicine, D-48149, Münster, Germany
3 Sequencing Core Facility, Max Planck Institute for Molecular Biomedicine, D-48149, Münster, Germany
4 Flow Cytometry Unit, Max Planck Institute for Molecular Biomedicine, D-48149, Münster, Germany
5 Department of Biology, Stanford University, Stanford, CA, United States
6 Institute for Stem Cell Biology and Regenerative Medicine, Stanford University School of Medicine, Stanford, CA, United States
7 Howard Hughes Medical Institute, Stanford, CA, United States

[#] Authors for correspondence:
Mara E. Pitulescu, PhD
Department of Tissue Morphogenesis
Max-Planck-Institute for Molecular Biomedicine, D–48149 Münster, Germany
Phone: +49 251 70365 425, E-Mail: mara.pitulescu@mpi-muenster.mpg.de

Ralf H. Adams, PhD
Department of Tissue Morphogenesis
Max-Planck-Institute for Molecular Biomedicine, D–48149 Münster, Germany
Phone: +49 251 70365 400, E-Mail: ralf.adams@mpi-muenster.mpg.de

Supplementary Figure 1: Common and distinct roles of EphB4 and ephrin-B2 in the retinal angiogenesis.

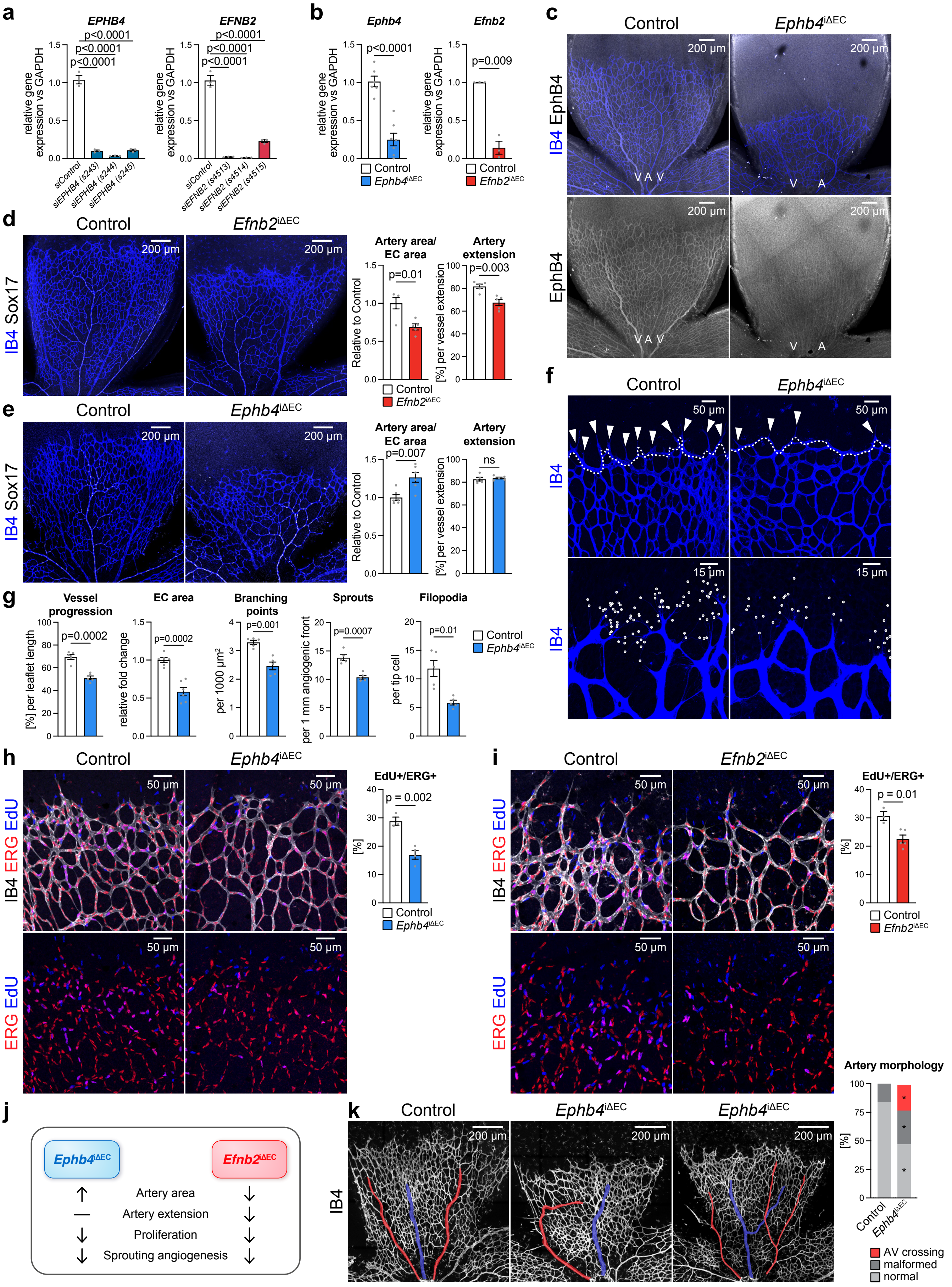

## Supplementary Figure 1: Common and distinct roles of EphB4 and ephrin-B2 in the retinal angiogenesis.

(**a**) Validation of *siEPHB4* and *siEFNB2* efficiency by RT-qPCR for 3 different siRNAs (n=3 experiments). (**b**) Validation of *Ephb4* and *Efnb2* gene deletion using *Cdh5CreERT2*. RT-qPCR of FACS sorted retinal ECs (n=6 control and 7 *Ephb4*$^{i\Delta EC}$ and n=3 control and 3 *Efnb2*$^{i\Delta EC}$ mice). (**c**) Confirmation of *Ephb4* inactivation. IB4 and EphB4 staining of control and *Ephb4*$^{i\Delta EC}$ retinas. (**d**, **e**) Confocal images of isolectin B4 (IB4) and Sox17 stained P6 retinal vessels (n=5 control and 5 *Efnb2*$^{i\Delta EC}$ mice (**d**) and n=6 control and 6 *Ephb4*$^{i\Delta EC}$ mice (**e**)). Quantitation of arterial area per total EC area and of artery extension defined by Sox17 arterial immunosignal. (**f**, **g**) EphB4 is required for sprouting angiogenesis. High magnification pictures of IB4 stained retinas (**f**). Graphs show quantitation of vessel progression (n=5 control and *Ephb4*$^{i\Delta EC}$), EC area (n=6 mice), branching points (n=5 mice), number of sprouts (n=5 mice) and filopodia per angiogenic front line (n=5 mice) (**g**). (**h**, **i**) Reduced proliferation of ECs in pan-endothelial *Ephb4* and *Efnb2* mutants. Graphs show quantitation of EdU+ per total ERG+ ECs (n=3 control and 4 *Ephb4*$^{i\Delta EC}$ and n=3 control and 5 *Efnb2*$^{i\Delta EC}$ mice). (**j**) Scheme summarizes vascular changes in mutant retinas. (**k**) Representative images of *Ephb4*$^{i\Delta EC}$ retina with malformed arteries and arteriovenous crossings compared to control. Graph shows quantitation of malformed arteries and normal vascular morphology (n= 19 control and 17 *Ephb4*$^{i\Delta EC}$). *P* values were calculated by one-way ANOVA (**a**), two-tailed unpaired *t*-test (**b**, **d**, **e**, **g**, **h**, **i**) and Chi-square test (**k**). *In vivo* experiments were performed with tamoxifen injections at P1-P3 following analysis at P6 (**c**, **d**, **e**, **f**, **h**, **i**, **k**). Data are presented as mean values +/- SEM. Source data are provided as a Source Data file.

Supplementary Figure 2: Tip cell deletion of *Ephb4* and *Efnb2*.

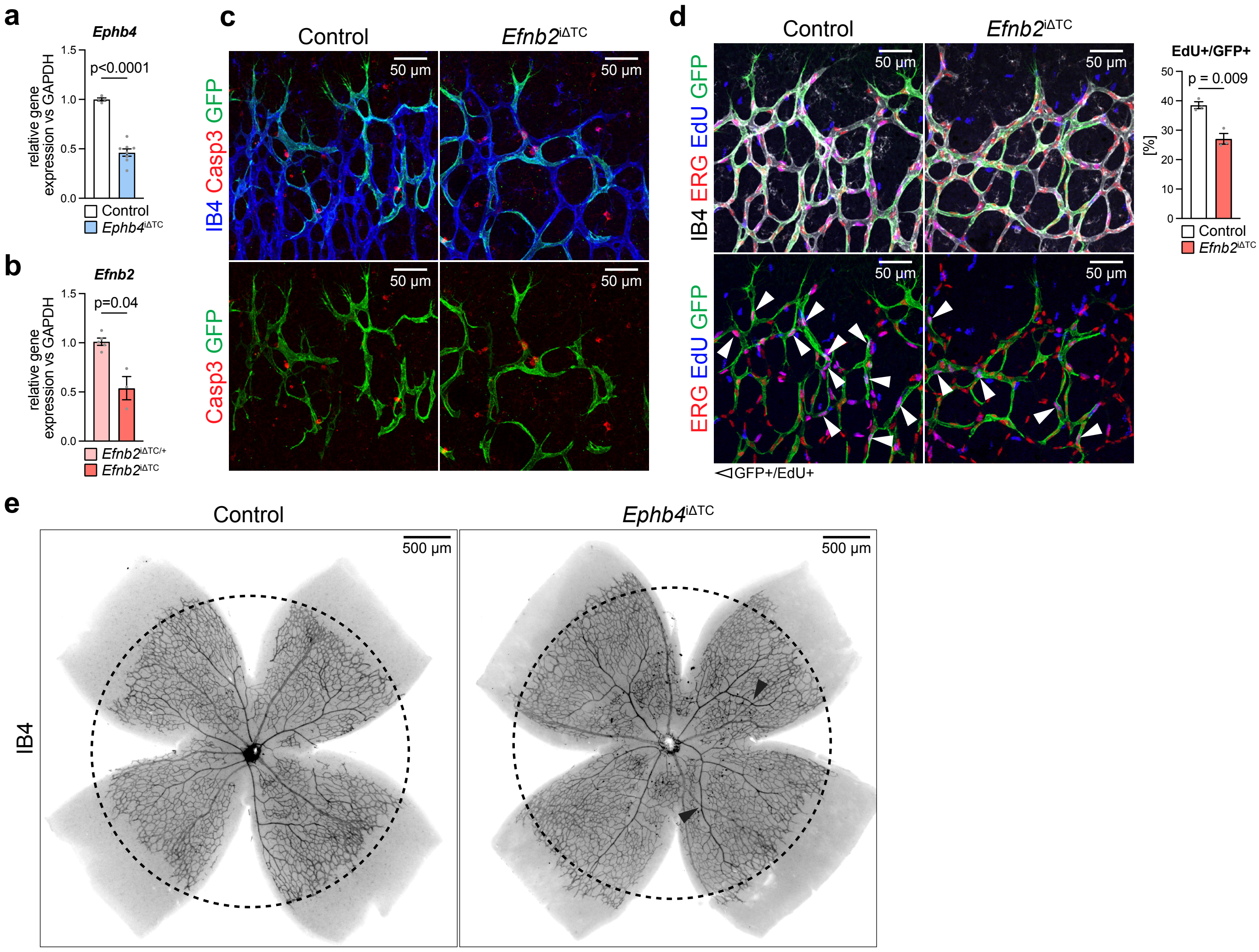

## Supplementary Figure 2: Tip cell deletion of *Ephb4* and *Efnb2*.

(**a**, **b**) Validation of *Ephb4* and *Efnb2* gene inactivation using *Esm1-CreERT2*. RT-qPCR of FACS sorted GFP+ retinal ECs (n=4 control and 8 *Ephb4*$^{i\Delta TC}$ and n=5 control (*Efnb2*$^{i\Delta TC/+}$) and 3 *Efnb2*$^{i\Delta TC}$ mice). (**c**) Confocal images of control and *Efnb2*$^{i\Delta TC}$ P6 retinas showing IB4+, GFP+ and Casp3+ cells. Note that there are no GFP+/Casp3+ ECs (n=3 mice). (**d**) EC proliferation (arrowheads) is reduced in *Efnb2* depleted tip cell progeny. Confocal images and quantitation of proliferating GFP+ ECs using IB4, ERG, EdU and GFP immunostaining (n=3 control and 3 *Efnb2*$^{i\Delta TC}$ mice). (**e**) IB4-stained overview pictures of control and *Ephb4*$^{i\Delta TC}$ mouse retinas. Arrowheads point to AV crossings. *P* values were calculated using two-tailed unpaired *t*-test (**a**, **b**, **d**). *In vivo* experiments were performed with tamoxifen injections at P1-P3 following analysis at P6 (**c**, **d**, **e**). Data are presented as mean values +/- SEM. Source data are provided as a Source Data file.

Supplementary Figure 3: Ephrin-B2 represses EphB4 expression but promotes VEGFR2 signaling.

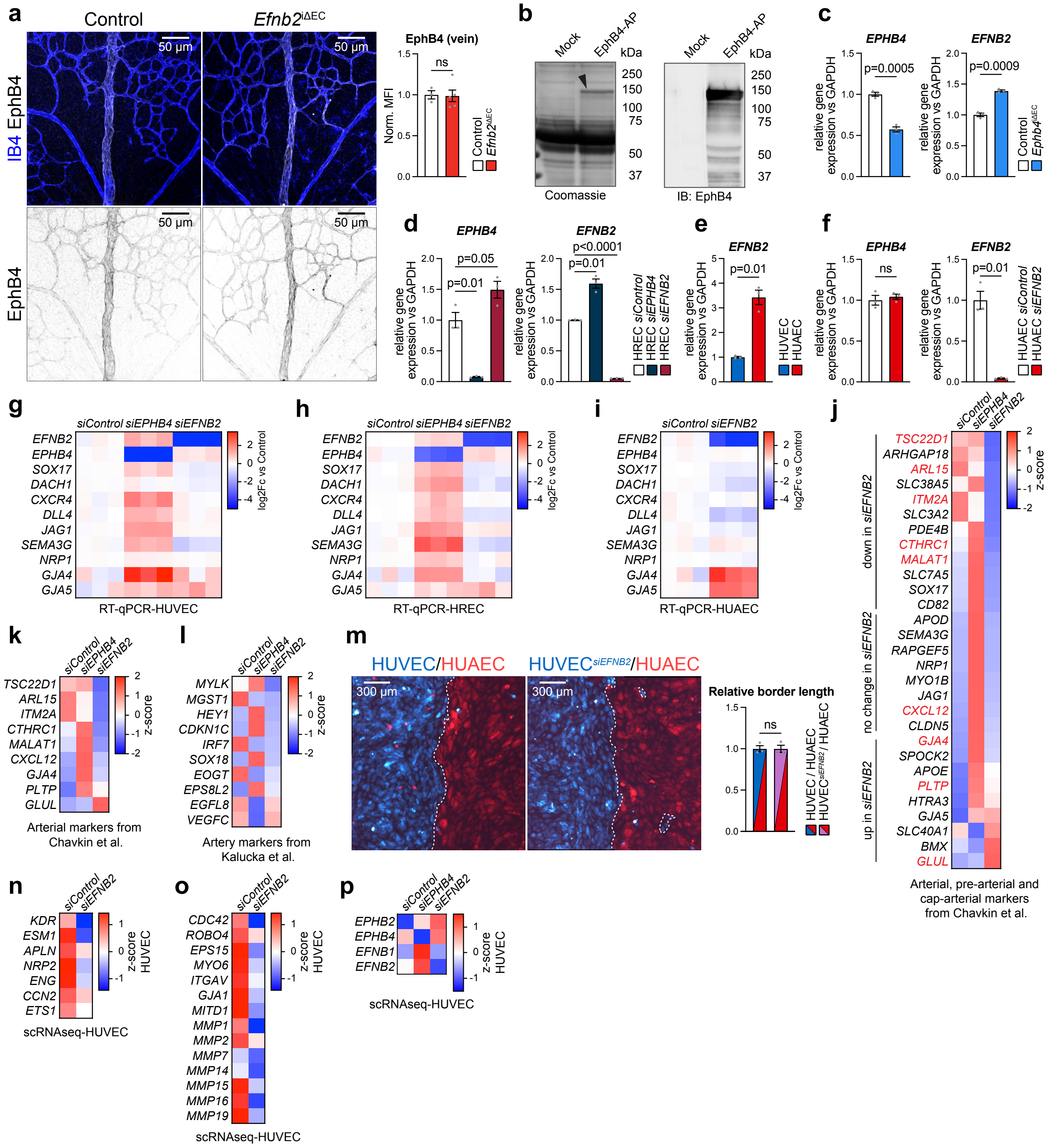

## Supplementary Figure 3: Ephrin-B2 represses EphB4 expression but promotes VEGFR2 signaling.

(**a**) EphB4 is unchanged in *Efnb2*$^{i\Delta EC}$ mature retinal vessels and central capillary plexus. EphB4 staining and immunosignal quantitation (n=3 control and 5 *Efnb2*$^{i\Delta EC}$ mice). (**b**) EphB4-AP protein expression. Coomassie blue and immunoblot detection of overexpressed EphB4-AP protein in HEK293 cell supernatant. (**c**) RT-qPCR of indicated genes in FACS-isolated P6 brain ECs (n=3 control and 3 *Ephb4*$^{i\Delta EC}$). (**d**, **e**, **f**) RT-qPCR of *EPHB4* and *EFNB2* in primary human retinal ECs (HRECs) upon *EPHB4* or *EFNB2* depletion (**d**), in HUVECs and HUAECs (**e**) and upon knockdown of *EFNB2* in HUAECs (**f**) (n=3 experiments). (**g**, **h**, **i**) RT-qPCR of indicated genes upon *EPHB4* and *EFNB2* knockdown in HUVECs (**g**) and HRECs (**h**) and upon knockdown of *EFNB2* in HUAECs (**i**) (n=3 experiments). (**j**, **k**, **l**) Comparative expression analysis of top arterial marker genes from P6 postnatal retina (Chavkin et al.[52]) and adult mouse brain (Kalucka et al.[53]) dataset, respectively, in *siControl*, *siEPHB4* and *siEFNB2* HUVEC scRNAseq own dataset. Z-score for tip cell-like main cluster was calculated in (**j**) for arterial (marked in red), pre-arterial and cap-arterial marker genes and in (**k**) only for arterial marker genes from Chavkin et al. retina dataset[52], while in (**l**) for arterial marker genes from Kalucka et al. brain dataset[53]. (**m**) Confrontation assay for heterotypic co-culture of HUVECs and HUAECs, as indicated. Final snapshots of CellTracker-labeled cells imaged for 48 h after removal of Ibidi insert. Quantitation graph for relative border length (n=3 experiments). (**n, o**) Decreased expression of genes related to angiogenesis (**n**) and cell migration (**o**) upon *EFNB2* KD in HUVECs compared to control. Genes selected from pseudobulk DGE analysis comparing *siControl* and *siEFNB2* in tip cell-like main cluster. (**p**) EphB and ephrin-B family genes are differentially affected by KD of EphB4 and ephrin-B2 in HUVECs. Results are based on pseudobulk DGE analysis comparing *siControl*, *siEPHB4* and *siEFNB2* in tip cell-like main cluster. *P* values were calculated using two-tailed unpaired *t*-test (**a**, **c-i, m**). *In vivo* experiments were performed with tamoxifen injections at P1-P3 following analysis at P6 (**a**). Data are presented as mean values +/- SEM. Source data are provided as a Source Data file.

Supplementary Figure 4: Regulation of *EFNB2* and *EPHB4* expression in HUVECs.

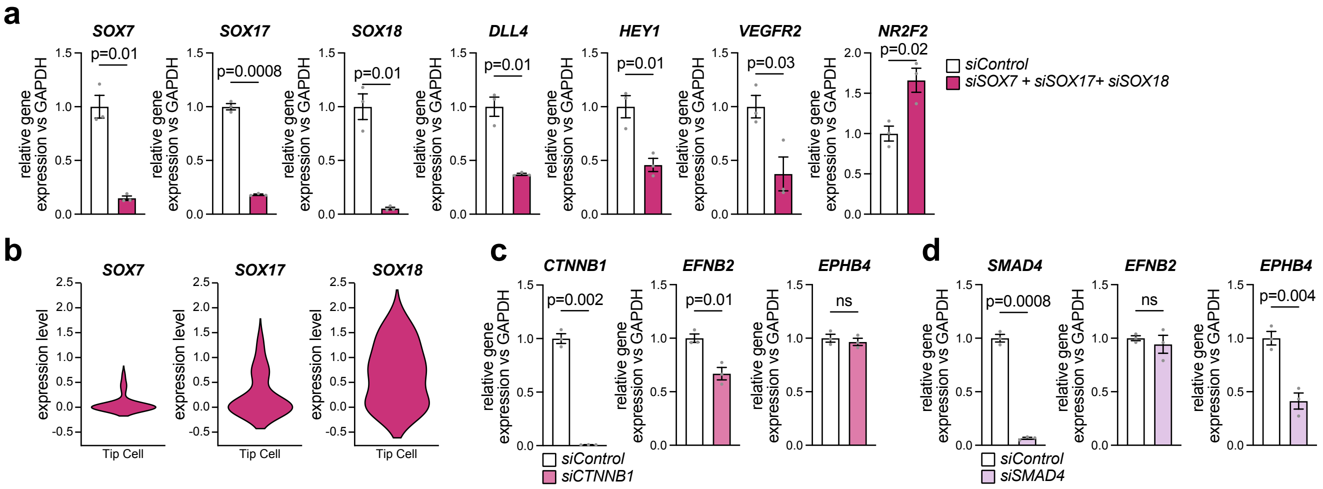

## Supplementary Figure 4: Regulation of *EFNB2* and *EPHB4* expression in HUVECs.

(**a**) SoxF factors promote arterial and repress venous gene expression. Validation by RT-qPCR of *SOX7* + *SOX17* + *SOX18* triple knockdown in HUVECs (n=3 experiments). (**b**) Violin plots of *SOX7*, *SOX17* and *SOX18* expression in P6 retinal tip cells. Data extracted from published scRNA-seq results[52]. (**c**, **d**) Not all transcription factors involved in vascular development and AV specification mediate reciprocal regulation of *EPHB4* and *EFNB2*. Graphs show RT-qPCR analysis of *siCTNNB1* vs *siControl* HUVECs (**c**) and *siSMAD4* vs *siControl* HUVECs (**d**) (n=3 experiments). *P* values were calculated using two-tailed unpaired *t*-test (**a**, **c**, **d**). Data are presented as mean values +/- SEM. Source data are provided as a Source Data file.

Supplementary Figure 5: ERK1/2 signaling promotes arterial gene expression.

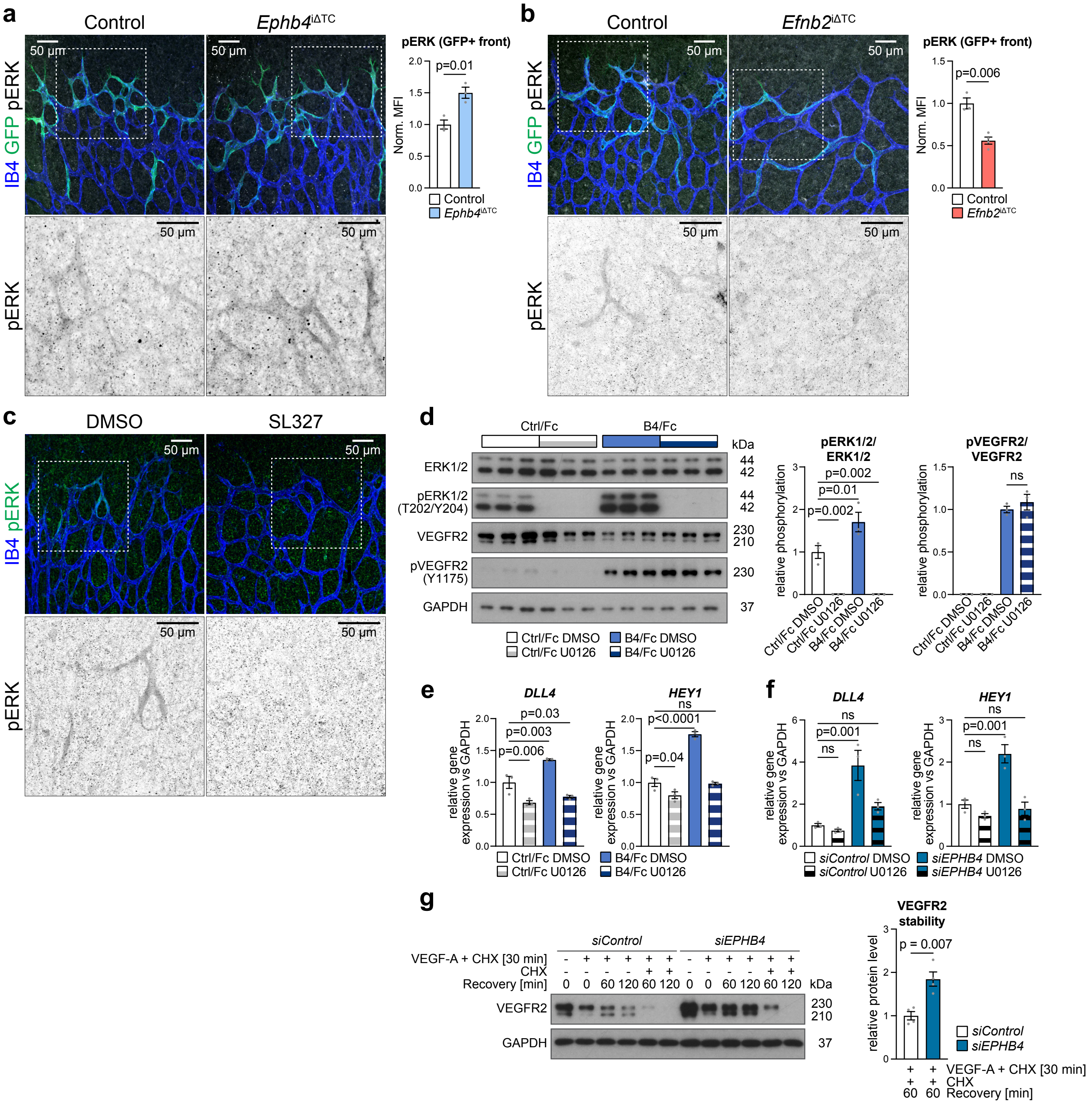

## Supplementary Figure 5: ERK1/2 signaling promotes arterial gene expression.

(**a**) Increased pERK1/2 (pERK) in *Ephb4*-deleted TC-derived progeny (n=3 control and 3 *Ephb4*$^{i\Delta TC}$). (**b**) Decreased pERK in *Efnb2*-deleted TC-derived progeny (n=3 control and 4 *Efnb2*$^{i\Delta TC}$). (**c**) Validation of pERK retinal immunosignal in mice treated with SL327 MEK inhibitor or vehicle. (**d**) VEGFR2 and ERK1/2 activation upon EphB4/Fc stimulation. Immunoblot for pVEGFR2 (Y1175) and pERK1/2 from stimulated HUVECs, treated with ERK inhibitor (U0126) or vehicle. Immunoblot quantitation of pVEGFR2 and pERK1/2 relative levels upon U0126 inhibitor or vehicle treatment (n=3 experiments). (**e**, **f**) ERK signaling is required downstream of ephrin-B2 stimulation (**e**) and *EPHB4* knockdown (**f**) to increase *DLL4* and *HEY1* expression. RT-qPCR for *HEY1* and *DLL4* (n=3 experiments). (**g**) Increased VEGFR2 stability in *EPHB4* KD cells. VEGFR2 immunoblotting of *siControl* and *siEPHB4* HUVECs pre-treated with VEGF-A and cycloheximide (CHX), washed and recovered in the absence or presence of CHX. Graph shows quantitation of VEGFR2 levels at 60 min of recovery (+ CHX) relative to 0 min (VEGF-A+CHX) (n=3 experiments). *P* values were calculated using two-tailed unpaired *t*-test (**a**, **b**, **g**) and one-way ANOVA (**d**, **e**, **f**). *In vivo* experiments were performed with 4-OHT injections at P4.5 following analysis at P6 (**a**, **b**). Data are presented as mean values +/- SEM. Source data are provided as a Source Data file.

Supplementary Figure 6: Notch and VEGF-A control arterial progenitors in the retina.

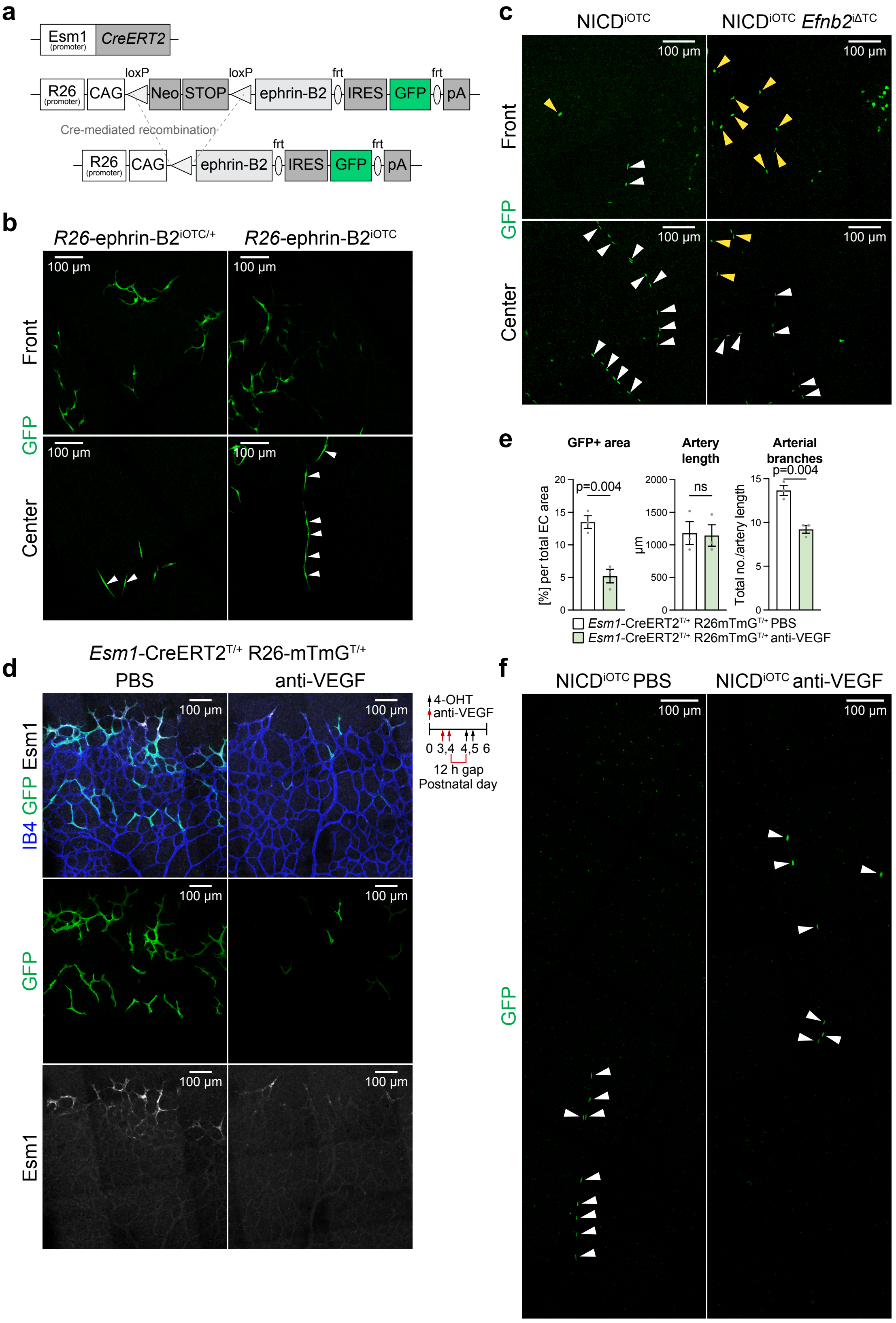

## Supplementary Figure 6: Notch and VEGF-A control arterial progenitors in the retina.

(**a**) Schematic depiction of the *Esm1-CreERT2* transgene and Cre-mediated ephrin-B2 overexpression. (**b**) Confocal images showing GFP+ ephrin-B2 overexpressing cells. (**c**) Confocal images of GFP+ (NICD+ cells) ECs in the $NICD^{iOTC}$ P6 retinal vasculature. (**d**) Induction of Esm1 expression in tip cells requires VEGF signaling. Confocal images of vehicle or anti-VEGF treated *Esm1-CreERT2 R26*-$mTmG^{+/T}$ retinas showing IB4+, Esm1+ and GFP+ (Esm1-derived) ECs. (**e**) Quantitation of GFP+ area per total EC area, artery length and arterial branches (n=3 vehicle and n=3 anti-VEGF). (**f**) Confocal images of vehicle or anti-VEGF treated $NICD^{iOTC}$ retinas showing GFP+ (NICD+) ECs. *P* values were calculated using two-tailed unpaired *t*-test (**c**). *In vivo* experiments were performed with tamoxifen injections at P1-P3 (**b**, **c**), 4-OHT injections at P4-P5 following analysis at P6 (**d**) or 4-OHT injections at P3-P4 followed by anti-VEGF injections at P5-P6 and analysis at P7 (**f**). Data are presented as mean values +/- SEM. Source data are provided as a Source Data file.

Supplementary Figure 7: Interactions between EphB4 and Notch during artery formation.

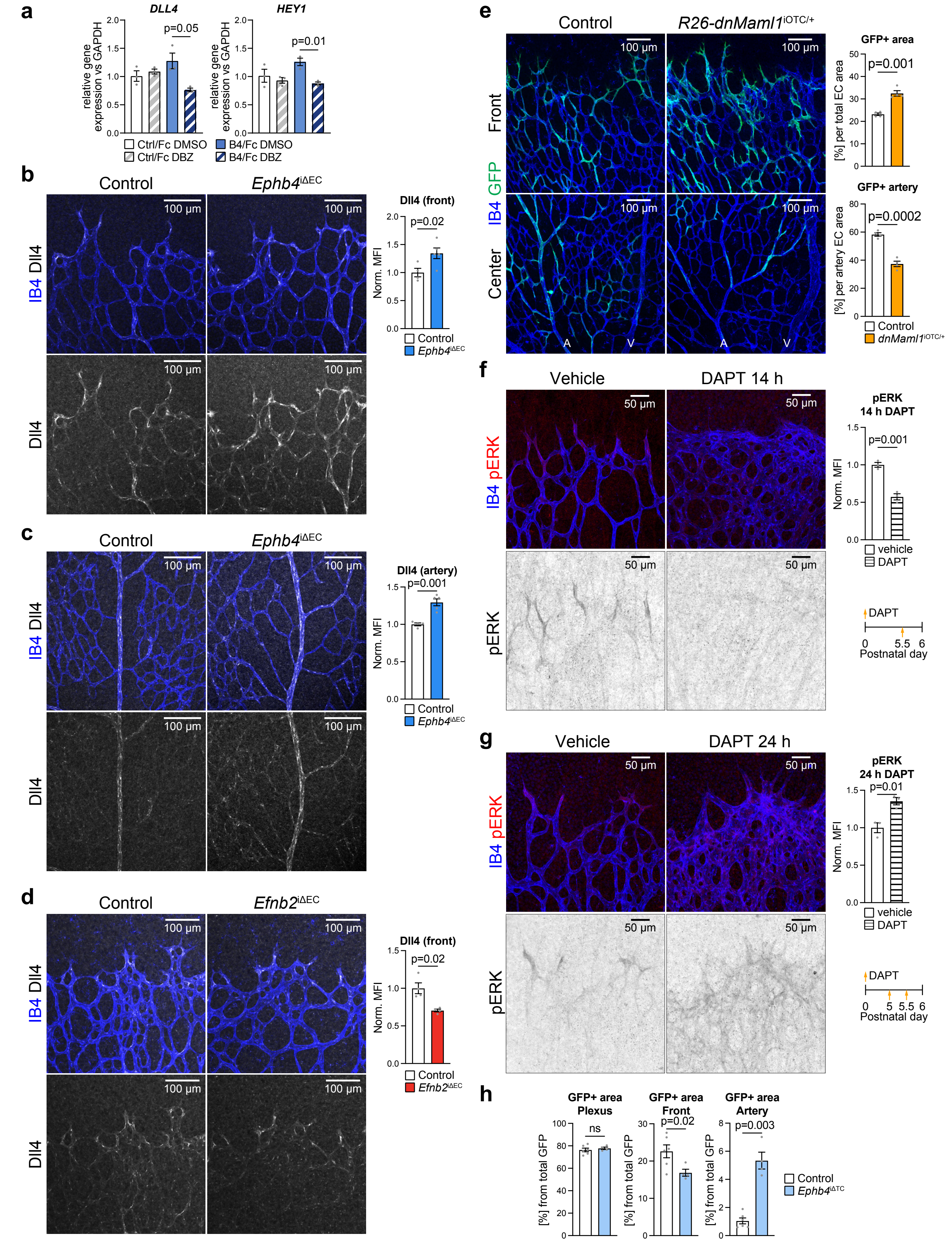

## Supplementary Figure 7: Interactions between EphB4 and Notch during artery formation.

(**a**) Normalization of *DLL4* and *HEY1* expression upon DBZ treatment of EphB4/Fc stimulated cells (n=3 experiments). (**b**, **c**) Quantitation of Dll4 immunosignal in the P6 control (n=4 animals) and *Ephb4*$^{i\Delta EC}$ (n= 5) vascular front (**b**) and retinal arteries (**c**). (**d**) Decreased Dll4 capillary plexus expression upon EC-specific *Efnb2* inactivation. Quantitation of Dll4 MFI in the retinal vascular front area (n=4 control and 4 *Efnb2*$^{i\Delta EC}$). (**e**) dnMaml1 overexpression in tip cells impairs artery growth. Graphs show quantitation of total GFP+ area and GFP+ arterial area (n=4 control and 4 *R26*-dnMaml1$^{iOTC/+}$). (**f**) Acute Notch inhibition (DAPT) for 14 h reduces pERK1/2. Graph shows quantitation of pERK MFI in angiogenic front area (n=3 vehicle and n=3 DAPT injected in WT C57BL6 mice). (**g**) Robust Notch inhibition (DAPT) for 24 h increased pERK. Graph shows quantitation of pERK MFI in angiogenic front area (n=3 vehicle and n=3 DAPT injected in WT C57BL6 mice). (**h**) Graphs showing the distribution of GFP+ area in capillary plexus, front and artery per total GFP positive area (n=6 control and 4 *Ephb4*$^{i\Delta TC}$ mice). *P* values were calculated by one-way ANOVA (**a**) and two-tailed unpaired *t*-test (**b**, **c**, **d**, **e**, **f, g, h**). *In vivo* experiments were performed with tamoxifen injections at P1-P3 following analysis at P6 (**b**, **c**, **d**, **e**). DAPT or vehicle was administered once, 14 h prior to analysis (**f**) or twice, at 24 h and 14 h prior to analysis (**g**). Data are presented as mean values +/- SEM. Source data are provided as a Source Data file.

Supplementary Figure 8: EphB4 and ephrin-B2 in cell cycle regulation.

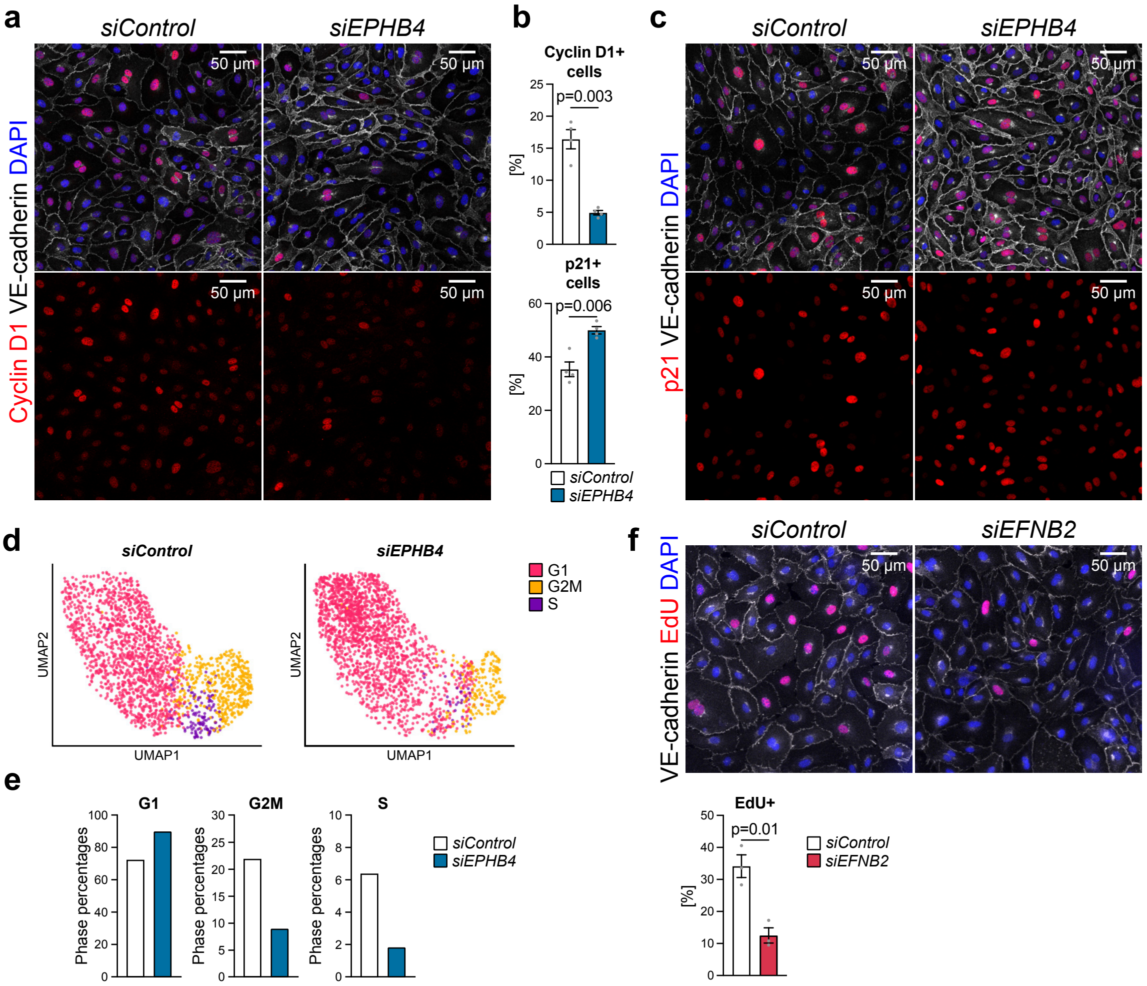

## Supplementary Figure 8: EphB4 and ephrin-B2 in cell cycle regulation.

(**a**-**c**) Decreased Cyclin D1 and increased P21 protein expression in *siEPHB4* cells at 48 h of knockdown (n=3 experiments). (**d**, **e**) *siEPHB4* cells show impaired cell cycle progression, as indicated by 2D UMAP representation of cell cycle phase distribution relative to *siControl* cells (**d**). Graphs represent proportion of cells in G1, G2/M and S phases (**e**). (**f**) Reduced proliferation of HUVECs after *EFNB2* KD. Graphs shows quantitation of EdU+ per total cells. *P* values were calculated by two-tailed unpaired *t*-test (**b**, **f**). Data are presented as mean values +/- SEM. Source data are provided as a Source Data file.

Supplementary Figure 9: EphB4, ephrin-B2 and Dach1 expression levels correlate with EC nuclear morphology and cell migration behavior under flow.

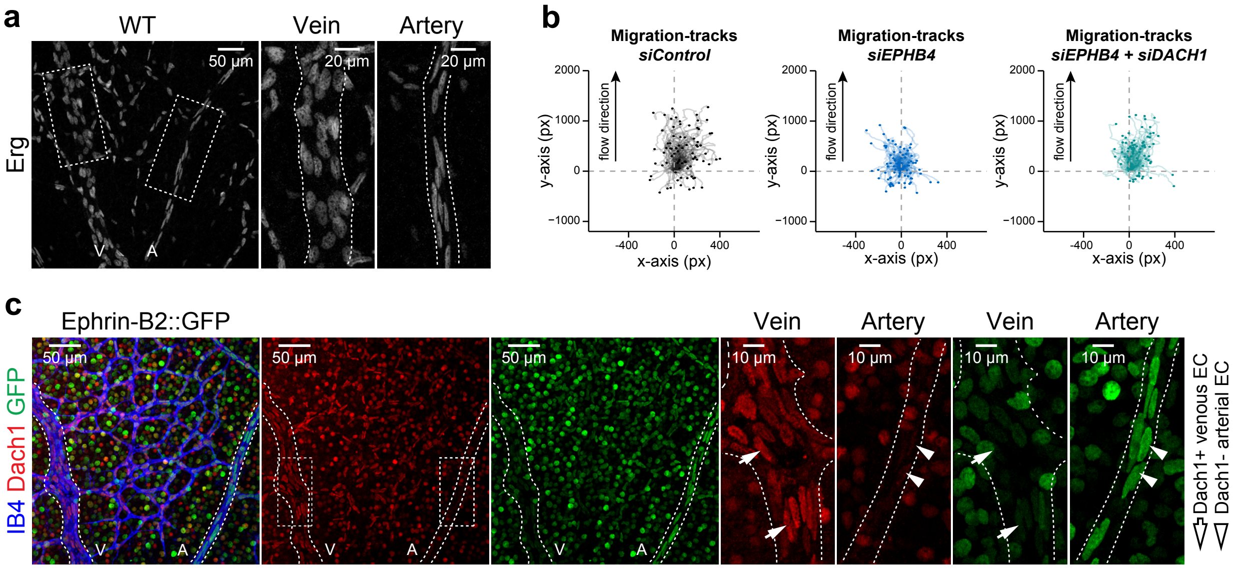

## Supplementary Figure 9: EphB4, ephrin-B2 and Dach1 expression levels correlate with EC nuclear morphology and cell migration behavior under flow.

(**a**) Arterial ECs align better to flow than venous cells. Example of wild-type (WT) P6 retinal vasculature immunostained for ERG. Boxed areas show representative parts of artery (A) or vein (V). (**b**) Examples of quantified migration tracks for *siControl*, *siEPHB4* and *siEPHB4/ DACH1* cells (80 randomly selected tracks/experiment). (**c**) Dach1 is expressed in retinal veins but not in arteries. IB4, GFP and Dach1 staining of the P6 *Efnb2-H2B-GFP* knock-in reporter retinal vasculature. Source data are provided as a Source Data file.

**Supplementary Table S1.** **Summary of genetic mouse models used in this study**

| Full name | Short name | Source/Reference |
|---|---|---|
| *Rosa26-mT/mG* | *R26-mTmG* | Muzumdar et al. 2007 |
| *Ephb4*$^{lox/lox}$ | / | Wang et al. 2015 |
| *Efnb2*$^{lox/lox}$ | / | Grunwald et al. 2004 |
| *Cdh5(PAC)-CreERT2* | *Cdh5-CreERT2* | Wang et al. 2010 |
| *Esm1-CreERT2* | | Rocha et al. 2014 |
| *Gt(ROSA)*$^{26Sortm1(Notch1)dam}$ | *NICD* | Murtaugh et al. 2003 |
| *Efnb2-GFP* | | Davy and Soriano, 2007 |
| C57BL/6J | WT | Janvier Labs |
| *Rosa26-ephrin-B2-GFP* | *R26-ephrin-B2-GFP* | this study |
| *Rosa26-Dach1OE* | *R26-Dach1* | Raftrey et al. 2021 |
| *Gt(ROSA)*$^{26Sortm1(MAML1)Wsp}$ | *R26-dnMaml1* | Tu et al. 2005 |

| Control genotype | Mutant genotype | Tamoxifen treatment | Figure |
|---|---|---|---|
| *Efnb2*$^{lox/lox}$ | *Efnb2*$^{lox/lox}$, *Cdh5(PAC)-CreERT2*$^{+/T}$ | P1-P3 50 µg | 2d, 3c, S1d, S1i, S3a, S7d |
| *Ephb4*$^{lox/lox}$ | *Ephb4*$^{lox/lox}$, *Cdh5(PAC)-CreERT2*$^{+/T}$ | P1-P3 50 µg | 2f, 3a, 4c, 5d, S1c, S1e, S1f, S1h, S1k, S7b, S7c |
| *Rosa26-mT/mG*$^{lox/+}$, *Esm1-CreERT2*$^{+/T}$ | *Ephb4*$^{lox/lox}$, *Rosa26-mT/mG*$^{lox/+}$, *Esm1-CreERT2*$^{+/T}$ | P1-P3 50 µg | 1c, 1g, 1i, 6b, 7a, S2e |
| *Rosa26-mT/mG*$^{lox/+}$, *Esm1-CreERT2*$^{+/T}$ | *Efnb2*$^{lox/lox}$, *Rosa26-mT/mG*$^{lox/+}$, *Esm1-CreERT2*$^{+/T}$ | P1-P3 50 µg | 1d, 1h, 7b, S2c, S2d |
| *NICD*$^{lox/lox}$, *Esm1-CreERT2*$^{+/T}$ | *Efnb2*$^{lox/lox}$, *NICD*$^{lox/lox}$, *Esm1-CreERT2*$^{+/T}$ | P1-P3 50 µg | 5b |
| *R26-ephrin-B2-GFP*$^{lox/+}$, *Esm1-CreERT2*$^{+/T}$ | *R26-ephrin-B2-GFP*$^{lox/lox}$, *Esm1-CreERT2*$^{+/T}$ | P1-P3 50 µg | 5a |
| *Ephb4*$^{lox/lox}$, *Rosa26-mT/mG*$^{lox/+}$, *Esm1-CreERT2*$^{+/T}$ | *Ephb4*$^{lox/lox}$, *R26-dnMaml1*$^{lox/+}$, *Rosa26-mT/mG*$^{lox/+}$, *Esm1-CreERT2*$^{+/T}$ | P1-P3 50 µg | 6c |
| *Rosa26-mT/mG*$^{lox/+}$, *Esm1-CreERT2*$^{+/T}$ | *Ephb4*$^{lox/lox}$, *Rosa26-mT/mG*$^{lox/+}$, *Esm1-CreERT2*$^{+/T}$ | P4.5 50 µg 4OHT | 6g, 9h, S5a |
| *Rosa26-mT/mG*$^{lox/+}$, *Esm1-CreERT2*$^{+/T}$ | *Ephb4*$^{lox/lox}$, *Rosa26-mT/mG*$^{lox/+}$, *Esm1-CreERT2*$^{+/T}$ | P4.5 50 µg 4OHT | 6h |
| *Rosa26-mT/mG*$^{lox/+}$, *Esm1-CreERT2*$^{+/T}$ | *Efnb2*$^{lox/lox}$, *Rosa26-mT/mG*$^{lox/+}$, *Esm1-CreERT2*$^{+/T}$ | P4.5 50 µg 4OHT | S5b |
| *Rosa26-mT/mG*$^{lox/+}$, *Esm1-CreERT2*$^{+/T}$ | *R26-dnMaml1*$^{lox/+}$, *Rosa26-mT/mG*$^{lox/+}$, *Esm1-CreERT2*$^{+/T}$ | P1-P3 50 µg | S7e |
| *Rosa26-mT/mG*$^{lox/+}$, *Esm1-CreERT2*$^{+/T}$ | *R26-Dach1*$^{lox/+}$, *Rosa26-mT/mG*$^{lox/+}$, *Esm1-CreERT2*$^{+/T}$ | P4.5 50 µg 4OHT | 9i |
| *NICD*$^{lox/lox}$, *Esm1-CreERT2*$^{+/T}$ vehicle | *NICD*$^{lox/lox}$, *Esm1-CreERT2*$^{+/T}$ anti-VEGF | P3, 4 50 µg 4OHT | 5c, S6d |
| *Rosa26-mT/mG*$^{lox/+}$, *Esm1-CreERT2*$^{+/T}$ | *R26-dnMaml1*$^{lox/+}$, *Rosa26-mT/mG*$^{lox/+}$, *Esm1-CreERT2*$^{+/T}$ | P4.5 50 µg 4OHT | 6e |
| *Rosa26-mT/mG*$^{lox/+}$, *Esm1-CreERT2*$^{+/T}$ vehicle | *Rosa26-mT/mG*$^{lox/+}$, *Esm1-CreERT2*$^{+/T}$ DAPT | P4.5 50 µg 4OHT | 6f |
| *Efnb2-GFP* | | / | 9c, S9c |
| *Efnb2-GFP, Esm1-CreERT2*$^{+/T}$, *Rosa26-RFP*$^{lox/+}$ | | P5 50 µg 4OHT | 9e |
| *Rosa26-mT/mG*$^{lox/+}$, *Esm1-CreERT2*$^{+/T}$ vehicle | *Rosa26-mT/mG*$^{lox/+}$, *Esm1-CreERT2*$^{+/T}$ anti-VEGF | P4, 5 50 µg 4OHT | S6b |

**Supplementary Table S2.   Antibodies used for retina immunostaining**

**a**

| Primary antibodies | Species | Clone | Company | Catalog # | Dilution |
|---|---|---|---|---|---|
| α-Dach1 | Rabbit | Polyclonal | Proteintech | 10914-1-AP | 1:200 |
| α-DLL4 | Goat | Polyclonal | R&D Systems | AF1389 | 1:50 |
| α-EphB4 | Goat | Polyclonal | R&D Systems | AF446 | 1:50 |
| α-ERG | Rabbit | Monoclonal | Abcam | ab110639 | 1:200 |
| α-GFP | Chicken | Polyclonal | Abcam | ab13970 | 1:1000 |
| α-GFP-AF-488 | Rabbit | Polyclonal | Invitrogen | A21311 | 1:500 |
| α-IB4-biotinylated | Griffonia simplicifolia | | Vector | B-1205 | 1:50 |
| α-pERK | Rabbit | Monoclonal | Cell Signaling | 4370 | 1:200 |
| α-Sox17 | Goat | Polyclonal | R&D Systems | AF1924 | 1:100 |
| α-Casp3 | Rabbit | Polyclonal | Cell Signaling | 9661 | 1:100 |

**b**

| Secondary antibodies | Species | Clone | Company | Catalog # | Dilution |
|---|---|---|---|---|---|
| α-chicken-AF-488 | Donkey | Polyclonal | Jackson ImmunoResearch | 703-545-155 | 1:500 |
| α-goat-AF-647 | Donkey | Polyclonal | Invitrogen | A21447 | 1:500 |
| α-goat-AF-647 | Donkey | Polyclonal | Thermo Scientific | A32849 | 1:500 |
| α-rabbit-AF-488 | Donkey | Polyclonal | Invitrogen | A21206 | 1:500 |
| α-rabbit-AF-546 | Donkey | Polyclonal | Invitrogen | A10040 | 1:500 |
| α-rabbit-AF-594 | Donkey | Polyclonal | Invitrogen | A21207 | 1:500 |
| Streptavidine-AF-405 | | | Invitrogen | S32351 | 1:100 |
| Streptavidine-AF-546 | | | Invitrogen | S11225 | 1:100 |

**Supplementary Table S3.** **Taqman probes used for qRT-PCR**

| Name/Gene | Target Species | Company | Assay ID/Cat# |
|---|---|---|---|
| CXCR4 FAM | Human | Thermo Fisher Scientific | Hs00607978_s1 |
| DACH1 FAM | Human | Thermo Fisher Scientific | Hs00974297_m1 |
| DLL1 FAM | Human | Thermo Fisher Scientific | Hs01011330_m1 |
| DLL4 FAM | Human | Thermo Fisher Scientific | Hs00184092_m1 |
| EFNB2 FAM | Human | Thermo Fisher Scientific | Hs00187950_m1 |
| EPHB4 FAM | Human | Thermo Fisher Scientific | Hs00174752_m1 |
| HEY1 FAM | Human | Thermo Fisher Scientific | Hs01114113_m1 |
| JAG1 FAM | Human | Thermo Fisher Scientific | Hs01070036_m1 |
| NR2F2 FAM | Human | Thermo Fisher Scientific | Hs00819630_m1 |
| SOX17 FAM | Human | Thermo Fisher Scientific | Hs00751752_s1 |
| SOX18 FAM | Human | Thermo Fisher Scientific | Hs00746079_s1 |
| SOX7 FAM | Human | Thermo Fisher Scientific | Hs00846731_s1 |
| VEGFR2 FAM | Human | Thermo Fisher Scientific | Hs00911700_m1 |
| GAPDH Endogenous Control VIC | Human | Thermo Fisher Scientific | 4326317E |
| SEMA3G FAM | Human | Thermo Fisher Scientific | Hs00928870_g1 |
| NRP1 FAM | Human | Thermo Fisher Scientific | Hs01546494_m1 |
| GJA4 FAM | Human | Thermo Fisher Scientific | Hs01098016_m1 |
| GJA5 FAM | Human | Thermo Fisher Scientific | Hs00979198_m1 |
| | | | |
| Ephb4 FAM | Mouse | Thermo Fisher Scientific | Mm01201156_m1 |
| Efnb2 FAM | Mouse | Thermo Fisher Scientific | Mm00438670_m1 |
| Gapdh Endogenous Control VIC | Mouse | Thermo Fisher Scientific | 4352339E |

**Supplementary Table S4. Antibodies used for western blotting**

**a**

| Primary antibodies | Species | Clone | Company | Catalog # | Dilution |
|---|---|---|---|---|---|
| α-Akt | Rabbit | Polyclonal | Cell Signaling | 9272 | 1:1000 |
| α-pAkt(S473) | Rabbit | Monoclonal | Cell Signaling | 4060 | 1:500 |
| α-bTAN20 | Rat | Monoclonal | DSHB | bTAN 20 | 1:700 |
| α-Dll4 | Rabbit | Polyclonal | Cell Signaling | 2589 | 1:1000 |
| α-EphB4 | Goat | Polyclonal | R&D Systems | AF446 | 1:2000 |
| α-Ephrin-B2 | Goat | Polyclonal | R&D Systems | AF496 | 1:2000 |
| α-Epsin1 | Rabbit | Monoclonal | Abcam | ab75879 | 1:1000 |
| α-ERK1/2 | Rabbit | Monoclonal | Cell Signaling | 4695 | 1:1000 |
| α-pERK1/2(T202/Y204) | Rabbit | Monoclonal | Cell Signaling | 4370 | 1:1000 |
| α-GAPDH | Rabbit | Monoclonal | Cell Signaling | 2118 | 1:10000 |
| α-Notch1 | Rabbit | Monoclonal | Cell Signaling | 4380 | 1:1000 |
| α-PLCy | Rabbit | Monoclonal | Cell Signaling | 5690 | 1:1000 |
| α-pPLCy(Y783) | Rabbit | Polyclonal | Cell Signaling | 2821 | 1:500 |
| α-Sox17 | Goat | Polyclonal | R&D Systems | AF1924 | 1:1000 |
| α-Tubulin | Mouse | Polyclonal | Sigma | T5168 | 1:6000 |
| α-VEGFR2 | Rabbit | Monoclonal | Cell Signaling | 2479 | 1:1000 |
| α-pVEGFR2(Y1175) | Rabbit | Monoclonal | Cell Signaling | 2478 | 1:500 |
| α-pVEGFR2(Y951) | Rabbit | Monoclonal | Cell Signaling | 4991 | 1:500 |

**b**

| Secondary antibodies | Species | Clone | Company | Catalog # | Dilution |
|---|---|---|---|---|---|
| α-goat HRP-linked | Bovine | Polyclonal | Jackson Immuno Research | 805-035-180 | 1:15000 |
| α-mouse HRP-linked | Sheep | | Amersham | NA931 | 1:40000 |
| α-rabbit HRP-linked | Goat | | Cell Signaling | 7074 | 1:15000 |
| α-rat-HRP-linked | Goat | | Amersham | NA935 | 1:15000 |

**Supplementary Table S5. Antibodies used for HUVEC immunostaining and FACS of murine ECs**

a

| Primary antibodies | Species | Clone | Company | Catalog # | Dilution |
|---|---|---|---|---|---|
| α-Dach1 | rabbit | polyclonal | Proteintech | 10914-1-AP | 1:200 |
| α-CyclinD1 | rabbit | monoclonal | Cell Signaling | 2978 | 1:50 |
| α-GOLPH4 | rabbit | polyclonal | Abcam | 28049 | 1:500 |
| α-p21 | rabbit | monoclonal | Cell Signaling | 2947 | 1:100 |
| α-Phalloidin | | | Invitrogen | A22287 | 1:40 |
| α-VE-Cadherin | rabbit | monoclonal | Cell Signaling | 3195 | 1:100 |
| α-VE-Cadherin | mouse | monoclonal | Santa Cruz | sc-9989 | 1:100 |
| α-VEGFR2 | goat | polyclonal | R&D Systems | AF644 | 1:100 |
| DAPI | | | Sigma | D9542 | 1:500 |

b

| Secondary antibodies | Species | Clone | Company | Catalog # | Dilution |
|---|---|---|---|---|---|
| α-goat-AF-488 | donkey | polyclonal | Invitrogen | A11057 | 1:500 |
| α-mouse-AF-488 | donkey | polyclonal | Invitrogen | A21202 | 1:500 |
| α-mouse-AF-546 | donkey | polyclonal | Invitrogen | A10036 | 1:500 |
| α-rabbit-AF-546 | donkey | polyclonal | Invitrogen | A10040 | 1:500 |
| α-rabbit-AF-647 | donkey | polyclonal | Invitrogen | A31573 | 1:500 |

c

| Conjugated primary antibodies | Species | Clone | Company | Catalog # | Dilution |
|---|---|---|---|---|---|
| α-CD45-BV421 | rat | monoclonal | Biolegend | 103134 | 1:100 |
| α-Ter119-BV605 | rat | monoclonal | Biolegend | 116239 | 1:100 |
| α-CD31-PerCP/ Cy5.5 | rat | monoclonal | Biolegend | 102420 | 1:50 |
| α-CD326-PE/Cy7 | rat | monoclonal | Biolegend | 118216 | 1:50 |
| α-CD140a-PE/Cy7 | rat | monoclonal | eBioscience | 25-1401-82 | 1:100 |
| α-CD140b-APC | rat | monoclonal | eBioscience | 17-1402-82 | 1:25 |
| α-Podoplanin-eFluor660 | hamster | monoclonal | eBioscience | 50-5381 | 1:100 |
| α-CD208-A647 | rat | monoclonal | Dentrics | DDX0192A647-100 | 1:50 |
