# Supplementary material for: Eph-ephrin signaling couples endothelial cell sorting and arterial specification
Source: Nat Commun. 2024 Apr 3;15:2539. doi: 10.1038/s41467-024-46300-0 (PMC10991410; doi:10.1038/s41467-024-46300-0)
Supplement: Supplementary file 3 — Description of Additional Supplementary Files [file 41467_2024_46300_MOESM3_ESM.pdf]

Description of Additional Supplementary Files

File Name: Supplementary Movie 1
Description: Migration of *siControl* HUVECs exposed to arterial flow. Migration tracks indicate cell migrating with (purple) or against flow (yellow).

File Name: Supplementary Movie 2
Description: Migration of *siEPHB4* HUVECs exposed to arterial flow. Migration tracks indicate cell migrating with (purple) or against flow (yellow).

File Name: Supplementary Movie 3
Description: Migration of *siEPHB4* + *siDACH1* HUVECs exposed to arterial flow. Migration tracks indicate cell migrating with (purple) or against flow (yellow).
