# Supplementary material for: Eph-ephrin signaling couples endothelial cell sorting and arterial specification
Source: Nat Commun. 2024 Apr 3;15:2539. doi: 10.1038/s41467-024-46300-0 (PMC10991410; doi:10.1038/s41467-024-46300-0)
Supplement: Supplementary file 7 — Reporting Summary [file 41467_2024_46300_MOESM7_ESM.pdf]

nature portfolio

Corresponding author(s): Mara E. Pitulescu and Ralf H. Adams

Last updated by author(s): Jan 30 2024

Reporting Summary

Nature Portfolio wishes to improve the reproducibility of the work that we publish. This form provides structure for consistency and transparency in reporting. For further information on Nature Portfolio policies, see our Editorial Policies and the Editorial Policy Checklist.

Please do not complete any field with "not applicable" or n/a. Refer to the help text for what text to use if an item is not relevant to your study.
For final submission: please carefully check your responses for accuracy; you will not be able to make changes later.

Statistics

For all statistical analyses, confirm that the following items are present in the figure legend, table legend, main text, or Methods section.

n/a	Confirmed	
☐	☒	The exact sample size (*n*) for each experimental group/condition, given as a discrete number and unit of measurement
☐	☒	A statement on whether measurements were taken from distinct samples or whether the same sample was measured repeatedly
☐	☒	The statistical test(s) used AND whether they are one- or two-sided<br>*Only common tests should be described solely by name; describe more complex techniques in the Methods section.*
☒	☐	A description of all covariates tested
☐	☒	A description of any assumptions or corrections, such as tests of normality and adjustment for multiple comparisons
☐	☒	A full description of the statistical parameters including central tendency (e.g. means) or other basic estimates (e.g. regression coefficient) AND variation (e.g. standard deviation) or associated estimates of uncertainty (e.g. confidence intervals)
☐	☒	For null hypothesis testing, the test statistic (e.g. *F*, *t*, *r*) with confidence intervals, effect sizes, degrees of freedom and *P* value noted<br>*Give P values as exact values whenever suitable.*
☒	☐	For Bayesian analysis, information on the choice of priors and Markov chain Monte Carlo settings
☒	☐	For hierarchical and complex designs, identification of the appropriate level for tests and full reporting of outcomes
☒	☐	Estimates of effect sizes (e.g. Cohen's *d*, Pearson's *r*), indicating how they were calculated

Our web collection on statistics for biologists contains articles on many of the points above.

Software and code

Policy information about availability of computer code

Data collection	Zeiss ZEN (black) (2012) was used to acquire images.
Data analysis	ImageJ2/Fiji was used for image processing and quantitation as described in the Methods section.<br>Statistical analysis was performed using GraphPad's Prism software (Prism 9) as described in the Methods section.<br>Python packages for scRNA-seq analysis: TrimGalore (v0.6.4); STAR (v2.7.3a); anndata (v0.8.0); gseapy (v0.10.7); harmonypy (v0.0.5); leidenalg (v0.8.8); matplotlib (v3.5.1); numpy (v1.21.5); pandas (v1.4.2); rpy2 (v3.4.5); scanpy (v1.8.2); scipy (v1.7.3); scrublet (v0.2.3); seaborn (v0.11.2) R packages for scRNA-seq analysis: scran (v1.22.1); SingleCellExperiment (v1.16.0); DESeq2 (v1.34.0); IHW (v1.22.0)<br>Analyses of scRNA-seq experiments have been performed in Python (3.8.2) and R (4.1.2) using the packages listed above. In addition, two Python libraries developed in house and project-specific analysis scripts are included in the provided code archive.<br>All code generated for data analysis of this study is deposited online in GitHub and can be accessed at https://github.com/Bioinformatics- Service-MPI-Munster/stewen-2024-nat-comms.

For manuscripts utilizing custom algorithms or software that are central to the research but not yet described in published literature, software must be made available to editors and reviewers. We strongly encourage code deposition in a community repository (e.g. GitHub). See the Nature Portfolio guidelines for submitting code & software for further information.

Data

Policy information about availability of data

All manuscripts must include a data availability statement. This statement should provide the following information, where applicable:

- Accession codes, unique identifiers, or web links for publicly available datasets
- A description of any restrictions on data availability
- For clinical datasets or third party data, please ensure that the statement adheres to our policy

The single-cell RNA-seq data generated in this study have been deposited in the Gene Expression Omnibus (GEO) under accession no. GSE223738. All other data supporting the findings of this study are available from the corresponding authors on reasonable request. Source data are provided with this paper.

Research involving human participants, their data, or biological material

Policy information about studies with human participants or human data. See also policy information about sex, gender (identity/presentation), and sexual orientation and race, ethnicity and racism.

Reporting on sex and gender	N/A
Reporting on race, ethnicity, or other socially relevant groupings	N/A
Population characteristics	N/A
Recruitment	N/A
Ethics oversight	N/A

Note that full information on the approval of the study protocol must also be provided in the manuscript.

Field-specific reporting

Please select the one below that is the best fit for your research. If you are not sure, read the appropriate sections before making your selection.

☒ Life sciences ☐ Behavioural & social sciences ☐ Ecological, evolutionary & environmental sciences

For a reference copy of the document with all sections, see nature.com/documents/nr-reporting-summary-flat.pdf

Life sciences study design

All studies must disclose on these points even when the disclosure is negative.

Sample size	Sample sizes of experiments were determined based on sample sizes from previous similar experiments (Pitulescu et al. Nature Cell Biology 2017) and sample availability.
Data exclusions	No data was excluded.
Replication	We performed at least 3 independent experiments/biological replicates. Experimental observations were reproducible and successful among the independent experiments.
Randomization	The work required no randomization. Mice were allocated into groups depending on their genotype. For inhibitor treatments, samples were subjected to different treatments and allocated in different groups accordingly.
Blinding	Blinding was not possible as genetic phenotype was evident from the image data.

Reporting for specific materials, systems and methods

We require information from authors about some types of materials, experimental systems and methods used in many studies. Here, indicate whether each material, system or method listed is relevant to your study. If you are not sure if a list item applies to your research, read the appropriate section before selecting a response.

Materials & experimental systems

n/a	Involved in the study	
☐	☒	Antibodies
☐	☒	Eukaryotic cell lines
☒	☐	Palaeontology and archaeology
☐	☒	Animals and other organisms
☒	☐	Clinical data
☒	☐	Dual use research of concern
☒	☐	Plants

Methods

n/a	Involved in the study	
☒	☐	ChIP-seq
☐	☒	Flow cytometry
☒	☐	MRI-based neuroimaging

Antibodies

Antibodies used: Reported in Supplementary Table 2, 4, 5

Validation: Anti-Dach1 (Proteintech 10914-1-AP) antibody has been validated by Proteintech by demonstrating immunostaining on HEK-293 cells. Anti-Dll4 (R&D Systems AF1389) antibody has been validated by R&D Systems by immunostaining on bEnd.3 mouse cells and by immunoblotting on bEnd.3 cell lysates. Anti-EphB4 (R&D Systems AF446) antibody has been validated by R&D Systems by immunostaining of fixed sections of mouse embryo. Further this antibody has been validated for immunoblotting in several publications including Luxan, et al. 2019 Elife. Anti-ERG (Abcam ab110639) antibody has been validated by Abcam by immunoblotting lysates of Jurkat, HT29 and MCF7 cells, as well as by immunostaining of embedded sections. Anti-GFP (Abcam ab13970) antibody has been validated by Abcam by immunostaining of transgenic mice expressing GFP. Conjugated anti-GFP-AF488 (Invitrogen A21311) has been validated by Invitrogen by immunoblotting of transfected HEK293 cell lysates. Further, this antibody has been validated for immunostaining in many publications including Leroy, et al. 2018 Nature, Castellano-Pozo, et al. 2020 Nature communications. Anti- IB4-biotinylated (Vector B-1205) has been validated for immunostaining in numerous publications including Gangadharan, et al. 2022 Nature, Fouani, et al. 2022 EMBO reports. Anti-pERK (Cell Signaling 4370) antibody has been validated by Cell Signaling Technology by immunoblotting of cell extracts of COS cells. Further, we validated the specificity of this antibody for immunostaining by treatment with SL327, an pERK inhibitor (see Supplementary Figure 3a). Anti-Sox17 antibody (R&D Systems AF1924) has been validated by the manufacturer by immunoblotting of human cell lysates, as well as by immunostaining of B16 mouse cell line. Anti-Akt (Cell Signaling 9272) antibody has been validated by Cell Signaling Technology by immunoblotting CHO cell lysates treated with non-targeted or Akt siRNA. Anti-pAkt (Cell Signaling 4060) has been validated by Cell Signaling Technology by immunoblotting of PC-3 cell extracts treated with or without LY294002 pAkt inhibitor. Anti-bTAN-20 (DSHB bTAN20) antibody has been validated for western blotting in several publications (Artavanis-Tsakonas, et al. 1995 PNAS, Kang 2017 Human molecular genetics). Anti-Dll4 (Cell Signaling 2589) antibody has been validated by Cell Signaling Technology by immunoblotting of HUVEC and COS cell extracts. Anti-Ephrin-B2 (R&D Systems AF496) antibody has been validated by R&D Systems by immunoblotting of mouse embryo tissue lysates. Anti-Epsin1 (Abcam ab75879) antibody has been validated by Abcam by immunoblotting of 293T, K562 and u87-MG cell lysates. Anti-ERK (Cell Signaling 4695) antibody has been validated by Cell Signaling Technology by immunoblotting of HeLa, NIH/3T3 and C6 cell lysates. Anti-GAPDH (Cell Signaling 2118) has been validated by the manufacturer by immunoblotting of various cell lysates. Anti-Notch1 (Cell Signaling 4380) has been validated by Cell Signaling Technology by immunoblotting of extracts from HPB-ALL and Molt4 cells. Anti-PLCy (Cell Signaling 5690) antibody has been validated by Cell Signaling Technology by immunoblotting of HeLa cell lysates treated with control or PLCy-targeting siRNA. Anti-pPLCy (Cell Signaling 2821) antibody has been validated by the manufacturer by immunoblotting cell lysates of NIH/3T3 cells untreated or treated with PDGF. Anti-Tubulin (Sigma T5168) anitbody has been validated by Sigma by immunoblotting various cell lysates. Anti-VEGFR2 (Cell Signaling 2479) antibody, as well as anti-pVEGFR2Y1175 (Cell Signaling 2478) antibody have been validated by the manufacturer by immunblotting of lysates generated from untreated or VEGF-stimulated HUVECs. Anti-pVEGFR2Y951 (Cell Signaling 4991) has been validated by Cell Signaling Technology by immunoblotting of lysates from untreated or phosphatase-treated HUVECs. Anti-CD45-BV421 (Biolegend 103134) antibody has been validated for FACS in several publications including Podd, et al. 2006 J Immunol., Haynes, et al. 2007 J Immunol. Anti-Ter119-BV605 (Biolegend) antibody has been validated for FACS in several publications (Heuser, et al. 2007 Blood, Chappaz, et al. 2007 Blood). Anti-CD31-PerCP/Cy5.5 (Biolegend 102420) antibody has been validated for FACS (Eilken, et al. 2017 Nature communications). Anti-CD326-PE/Cy7 (Biolegend 118216) antibody has been validated for FACS in the literature (Farr, et al. 1991 J. Histochem. Cytochem.). Anti-CD140a-PE/Cy7 (eBioscience 25-1401-82) antibody, as well as anti-CD140b-APC (eBioscience 17-1402-82) antibody have been validated by the manufacturer by staining and sorting of NIH/3T3 cells. Anti-Podoplanin-eFlour660 (eBioscience 50-5381) antibody has been validated by the manufacturer by staining and sorting of TE-71 cells.

Eukaryotic cell lines

Policy information about cell lines and Sex and Gender in Research

Cell line source(s): HUVECs (Human umbilical venous endothelial cells) (Thermo Fisher C0035C or provitro 1210111) HUAEC (Human umbilical arterial endothelial cells) (provitro 121 0112)
HUREC (Human retinal endothelial cells) (Innoprot P10880)

Authentication: None of the cell lines have been authenticated.

Mycoplasma contamination: Cell lines were not tested for mycoplasma contamination, but no indication of such contamination was observed.

Commonly misidentified lines (See ICLAC register): No commonly misidentified cell lines were used.

Animals and other research organisms

Policy information about studies involving animals; ARRIVE guidelines recommended for reporting animal research, and Sex and Gender in Research

Laboratory animals	A summary of all genetic mouse models used in this study can be found in supplementary table S1. To inactivate Ephb4 in the postnatal endothelium, Ephb4lox/lox mice111 and Cdh5(PAC)-CreERT2+/T transgenic mice43 were interbred. Male Ephb4lox/lox, Cdh5-CreERT2+/T offspring were mated with Ephb4lox/lox female mice to generate progeny for analysis. For Cre activation, pups were injected from P1 to P3 with 50 µg tamoxifen (Sigma,T5648), followed by analysis at P6.<br>For TC-specific Ephb4 loss-of-function experiments, mice homozygous for the R26-mTmG transgene48 were interbred with the Ephb4lox/lox line to generate Ephb4+/lox, R26-mTmGT/T offspring. Additionally, Ephb4lox/lox mice were interbred with Esm1-CreERT2+/T transgenic animals34 to generate Ephb4+/lox, Esm1-CreERT2+/T offspring. These mice were interbred with Ephb4+/lox, R26-mTmGT/T animals to obtain R26-mTmG+/T, Esm1-CreERT2+/T controls and Ephb4lox/lox, R26-mTmG+/T, Esm1-CreERT2+/T mutants. Pups were injected daily from P1 to P3 with 50 µg tamoxifen and analyzed at P6. For acute deletion experiments, pups were injected with a single dose of 50 µg 4-hydroxy-tamoxifen (4-OHT; Sigma, H7904) at P4.5 and analyzed at P6.<br>To achieve pan-endothelial and TC-specific deletion of Efnb2112, similar mating strategies as described above were used. Pups were injected with 50 µg tamoxifen from P1 to P3 and retinas analyzed at P6.<br>For TC-specific dnMaml1 overexpression experiments, we mated R26-dnMaml1+/T77 and R26-mTmGT/T, Esm1-CreERT2+/T mice. Offspring was daily injected from P1 to P3 with 50µg tamoxifen and analyzed at P6. For combined inactivation of Ephb4 and overexpression of dnMaml1 in TCs, Ephb4lox/lox, R26-dnMaml1+/T females were bred with Ephb4lox/lox, R26-mTmGT/T, Esm1-CreERT2+/T males. Cre activation was induced by daily injection of 50µg tamoxifen from P1 to P3. Retinas were analyzed at P6.<br>For combined TC-specific overexpression of active Notch and deletion of Efnb2, Gt(ROSA)26Sor tm1(Notch1)dam homozygous mice (NICDlox/lox)113 were interbred with Efnb2lox/lox, Esm1-CreERT2+/T. Further, NICDlox/lox, Efnb2+/lox, Esm1-CreERT2+/T male mice were crossbred with NICDlox/lox, Efnb2+/lox females, to obtain NICDlox/lox, Efnb2lox/lox, Esm1Cre-ERT2+/T double mutants and NICDlox/lox, Esm1-CreERT2+/T single mutants. Pups were injected daily from P1 to P3 with 50µg tamoxifen and retinas were analyzed at P6.<br>For overexpression of ephrin-B2 in TCs, Esm1-CreERT2+/T transgenics were combined with R26-ephrin-B2-GFPT/T mice. These mice carry a transcriptional stop cassette flanked by loxP sites, which is followed by the wild-type sequence of ephrin-B2 and an frt flanked IRES enhanced GFP (EGFP) construct. A map of the transgenic construct is shown in Supplementary Fig. 6a. R26-ephrin-B2-GFP+/T, Esm1-CreERT2+/T offspring were further bred with R26-ephrin-B2-GFPT/T, to obtain heterozygous and homozygous mutants for the R26-ephrin-B2-GFP allele in combination with Esm1-CreERT2. Pups were injected with 50 µg tamoxifen from P1 to P3. Heterozygous and homozygous mutant littermates were analyzed at P6.<br>TC-specific overexpression of Dach1 was achieved by interbreeding of R26-Dach1+/T (ref. 93) and R26-mTmGT/T, Esm1-CreERT2+/T mice. Pups were injected daily with 50µg tamoxifen from P1 to P3 and retinas were analyzed at P6.<br>For visualization of endogenous ephrin-B2 expression, Efnb2-GFP knock-in reporter mice55 were used. These mice carry a complementary DNA encoding a fusion construct of histone H2B and enhanced GFP, which has been inserted into the endogenous Efnb2 locus. As homozygous Efnb2-GFP mice are not viable, Efnb2-GFP+/T animals were interbred with C57BL/6J wild-type mice to obtain heterozygous offspring, which was analyzed at P6.<br>Lineage tracing experiments of Esm1-derived cells were performed using R26-mTmG+/T, Esm1-CreERT2+/T offspring generated by mating female mice homozygous for the R26-mTmG transgene with Esm1-CreERT2+/T males. Cre recombination was achieved by injection of single dose 4-OHT at P3 or P5, followed by analysis at P6.<br>Immunostaining of wild-type retinas was performed using C57BL/6J P6 pups.<br>For Notch inhibition, DAPT (Merck Millipore, 565770) was dissolved in 10% ethanol, 90% peanut oil and 0.1 mg/g body weight were intraperitoneally (IP) injected at P5.5 and pups were analyzed 14 hours later at P6. For robust Notch inactivation mice were injected at P5 twice, 24 hours and 14 hours prior analysis at P6. For vehicle treatment 10% ethanol, 90% peanut oil of similar volumes was used.<br>To inhibit ERK phosphorylation, SL327 (Selleckchem, S1066) was dissolved in 10% DMSO, 90% corn coil and IP injected into pups at a dosage of 120 mg/kg body weight 36 hours and 12 hours prior to dissection at P680.<br>To block VEGF, pups were IP injected with VEGF blocking antibody (Creative Biolabs, HPAB-0330CQ-F(E), clone mAb G6-31) using a dosage of 5 mg/kg body weight.<br>To assess EC proliferation, P6 pups were injected IP with EdU (100 µg) 90 min before dissection17. EdU-incorporated cells in the retina were detected using the Click-iT EdU Imaging Kit (Thermo Fisher Scientific, C10340).<br>All animal procedures were performed in compliance with the relevant laws and institutional guidelines, were approved by local animal ethics committees and were conducted at the MPI for Molecular Biomedicine with permissions granted by the Landesamt für Natur, Umwelt und Verbraucherschutz (LANUV) of North Rhine-Westphalia. Animals were combined in groups for experiments irrespective of their sex.
Wild animals	No wild animals were used.
Reporting on sex	Animals were not split across sex as phenotypes were applicable to both sexes.
Field-collected samples	No field-collected samples were used.
Ethics oversight	All animal experiments were performed according to the institutional guidelines and laws, approved by local animal ethical committee and were conducted at the Max Planck Institute for Molecular Biomedicine with necessary permissions (Az 81-02.04.2019.A114, Az 81-02.04.2022.A390) granted by the Landesamt für Natur, Umwelt und Verbraucherschutz (LANUV) of North Rhine-Westphalia, Germany.

Note that full information on the approval of the study protocol must also be provided in the manuscript.

Plants

Seed stocks	N/A
Novel plant genotypes	N/A
Authentication	N/A

Flow Cytometry

Plots

Confirm that:

☒ The axis labels state the marker and fluorochrome used (e.g. CD4-FITC).

☒ The axis scales are clearly visible. Include numbers along axes only for bottom left plot of group (a 'group' is an analysis of identical markers).

☒ All plots are contour plots with outliers or pseudocolor plots.

☒ A numerical value for number of cells or percentage (with statistics) is provided.

Methodology

Sample preparation	Reported in Methods section
Instrument	BD FACSAria IIIu (BD Biosciences)
Software	BD FACSDiva 8.0.1 and FlowJo 10.8.1 (BD Biosciences)
Cell population abundance	Cell abundance for a representative control:<br>cells/Single Cells/live CD45- cells/Ter119-/5x-CD31+ Freq. of Parent: 45,6<br>cells/Single Cells/live CD45- cells/Ter119-/5x-CD31+ Freq. of Grandparent: 18,1<br>cells/Single Cells/live CD45- cells/Terr119-/5x-CD31+ Freq. of live CD45- cells: 14,2
Gating strategy	In the initial FSC/SSC plot cells were separated from debris and aggregates followed by the exclusion of doublets. Subsequently cells negative for DAPI, CD45, Ter119, CD140a, EpCAM, CD140b, Podoplanin and CD208 were selected. From the resulting fraction CD31 or GFP positive cells were gated for sorting. For all fluorescence channels the threshold of positivity was determined using flourescence minus one (FMO)-controls.

☒ Tick this box to confirm that a figure exemplifying the gating strategy is provided in the Supplementary Information.

This checklist template is licensed under a Creative Commons Attribution 4.0 International License, which permits use, sharing, adaptation, distribution and reproduction in any medium or format, as long as you give appropriate credit to the original author(s) and the source, provide a link to the Creative Commons license, and indicate if changes were made. The images or other third party material in this article are included in the article's Creative Commons license, unless indicated otherwise in a credit line to the material. If material is not included in the article's Creative Commons license and your intended use is not permitted by statutory regulation or exceeds the permitted use, you will need to obtain permission directly from the copyright holder. To view a copy of this license, visit http://creativecommons.org/licenses/by/4.0/
